# Isolated Abducens Nerve Palsy in an Adolescent With Confounding Multisystem Serology: A Case Report and Diagnostic Review

**DOI:** 10.1155/crpe/2146062

**Published:** 2025-09-24

**Authors:** Roberto Paparella, Irene Bernabei, Fabiola Panvino, Camilla Ajassa, Lorenzo Benedetti, Lucia Leonardi, Alberto Spalice, Luigi Tarani

**Affiliations:** ^1^Department of Maternal Infantile and Urological Sciences, Sapienza University of Rome, Rome, Italy; ^2^Department of Human Neuroscience, Sapienza University of Rome, Rome, Italy; ^3^Department of Public Health and Infectious Diseases, Sapienza University of Rome, Rome, Italy

**Keywords:** abducens nerve palsy, adolescence, *Borrelia burgdorferi*, *Chlamydia pneumoniae*, herpes simplex virus, *Mycoplasma pneumoniae*, neuroborreliosis, ocular motility disorders, pediatric

## Abstract

Cranial nerve palsies in pediatric patients are rare and can be challenging to diagnose due to the broad spectrum of potential causes, including infections, inflammation, neoplasms, and idiopathic conditions. Abducens nerve palsy (ANP), though uncommon, is of particular interest due to its association with both intracranial and systemic pathologies. We present the case of a 16-year-old male who developed isolated left ANP of presumed infectious-inflammatory origin. Initial neurological and ophthalmological assessments revealed esotropia and marked abduction deficit without other cranial nerve involvement. Brain magnetic resonance imaging showed enhancement of the left abducens nerve consistent with neuritis, while cerebrospinal fluid analysis and initial laboratory investigations were unremarkable. Serological testing revealed low-positive IgM for *Mycoplasma pneumoniae*, *Chlamydia pneumoniae*, Herpes simplex virus (HSV)-1/2, and *Borrelia burgdorferi*, while polymerase chain reaction for HSV and Borrelia were negative. The patient was treated with corticosteroids, antibiotics, and antivirals, showing mild improvement in eye mobility, and follow-up imaging revealed resolution of the inflammatory changes. Despite persistent low IgM positivity in subsequent tests, the patient fully recovered within 6 months. Although the exact etiology remains unclear, the combination of clinical response and serological findings suggests a possible infectious or immune-mediated process. This case underscores the diagnostic complexity of pediatric ANP and highlights the importance of considering a broad differential diagnosis and using a multidisciplinary approach in management. Further research is needed to better understand the role of mild serological findings and improve diagnostic strategies for such conditions.

## 1. Introduction

Cranial nerve palsies in pediatric patients are rare and often pose a diagnostic challenge due to a wide range of potential etiologies, including infectious, inflammatory, neoplastic, vascular, and idiopathic causes [1]. Among these, abducens nerve palsy (ANP) is particularly noteworthy due to its association with both intracranial and systemic pathologies [2, 3]. The patient's age represents a key factor in identifying the underlying cause and deciding whether additional investigations, such as neurological imaging, are necessary [4]. Benign acquired isolated ANP is an uncommon condition in children, with recurrences being exceptionally rare. The diagnosis is established retrospectively, by ruling out other causes, following a sufficient period of careful monitoring [5].

We present the case of a previously healthy adolescent who developed an isolated left abducens nerve neuritis of presumed infectious-inflammatory origin, managed successfully with corticosteroid in association with antibiotic and antiviral therapy. Multiple low-positive IgM serologies for *Mycoplasma pneumoniae*, *Chlamydia pneumoniae*, Herpes simplex virus (HSV), and *Borrelia burgdorferi* added complexity to the diagnostic process, with their clinical significance remaining unclear despite the patient's eventual recovery.

## 2. Case Presentation

A 16-year-old male with an unremarkable medical history presented to our Emergency Department with a 2-day history of progressive diplopia. Familial, perinatal, and developmental history were unremarkable, and routine immunizations were up to date.

**Figure 1:**
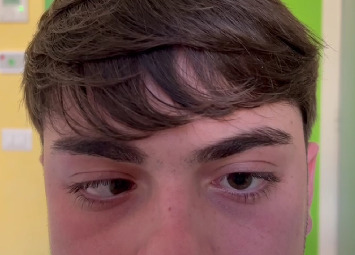
Marked limitation of abduction of the left eye during attempted leftward gaze, consistent with left abducens nerve palsy.

**Figure 2:**
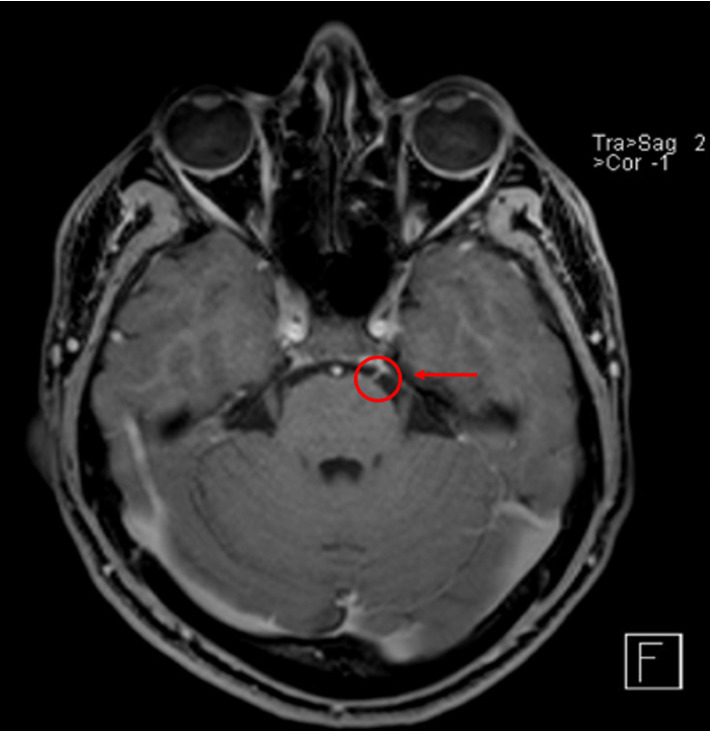
Brain MRI showing thickening and enhancement of the intracisternal segment of the left abducens nerve (red circle and arrow).

Neurological and ophthalmological assessments revealed an isolated left ANP (Figure 1) with esotropia of the left eye in primary position and marked abduction deficit; no signs or symptoms of any other cranial nerve involvement were observed. Magnetic resonance imaging (MRI) and angiography of the brain revealed the thickening and enhancement of the intracisternal segment of the left abducens nerve (Figure 2), consistent with inflammatory or reactive neuritis, with no vascular anomalies and intracranial or orbital lesions. Cerebrospinal fluid (CSF) analysis was negative for infection, malignancy, and autoimmune markers, with a normal opening pressure at lumbar puncture. Initial laboratory investigations, including serum inflammatory markers, autoimmune screening, and a complete blood count, were normal.

The patient was admitted for further evaluation and treatment. High-dose intravenous methylprednisolone (1 g/day for 5 days) was initiated, followed by an oral prednisone taper. Serological testing revealed low-positive *Mycoplasma pneumoniae* and *Chlamydia pneumoniae* IgM (index value: 1.3 and 4.6, respectively, and positivity threshold: > 1.1) and low positive HSV-1/2 IgM (index value: 1.3 and positivity threshold: > 1.1), with positive HSV-1 IgG and negative HSV-1 IgG and blood polymerase chain reaction (PCR) for HSV-1/2. Concurrent antiviral (acyclovir 750 mg three times a day for 14 days) and antibiotic (azithromycin 500 mg once a day for 5 days) therapies were anyway started. Additional investigations ruled out malignancy and systemic infectious processes. A thrombophilia workup and vasculitis screening were conducted to rule out alternative causes of ischemic cranial neuropathy, with no abnormalities identified. Blood and CSF antiganglioside antibodies were negative.

Serological testing for *Borrelia burgdorferi* revealed low IgM positivity (index value: 1.16 and positivity threshold: > 1.1), while IgG titers and PCR testing for DNA were negative. Given these findings, neuroborreliosis was suspected. However, CSF analyses, including cell counts, protein levels, glucose, and PCR for Borrelia, were unremarkable. Infectious disease consultation recommended initiating doxycycline therapy (100 mg twice daily for 14 days). Given the diagnostic uncertainty and the simultaneous detection of multiple potential pathogens by serology, a broad empirical regimen was therefore initiated, including antivirals, antibiotics targeting atypical bacteria, and corticosteroids to address a possible postinfectious inflammatory neuritis.

During hospitalization, mild clinical improvement in eye mobility was noted, and follow-up MRI showed resolution of inflammatory changes. Visual evoked potentials revealed a slight delay in left eye latency, consistent with residual neural inflammation. The patient was discharged with a corticosteroid taper, multivitamin supplementation, and scheduled follow-up in neurology and ophthalmology.

Serial ophthalmological evaluations revealed stable but unresolved deficits in ocular motility and persistent horizontal diplopia in all gaze directions. Further serological testing showed persistent, unchanged low IgM positivity with negative IgG titers and PCR test, raising questions regarding its clinical significance. At the most recent follow-up, 1 year after the initial admission, the patient fully recovered, with no long-term sequelae. Despite extensive investigations, the underlying etiology of the persistent ANP remained unclear.

## 3. Discussion

Cranial nerve palsies in pediatric patients are rare, and their etiology often poses significant diagnostic challenges due to the broad spectrum of potential causes, including infectious, inflammatory, vascular, and idiopathic conditions [1, 6]. Among these, ANP is a clinically relevant presentation and represents the second most common cranial nerve palsy in pediatric age with an incidence of 2.5 cases per 100,000 [7]. However, geographic factors may impact on the prevalence of cranial nerve palsies, resulting in regional differences in which nerve is most commonly affected. The involvement of the abducens nerve, responsible for the lateral rectus muscle, can result in eye misalignment, leading to diplopia and an impaired ability to move the eye laterally [8, 9]. In pediatric populations, ANP can arise from a variety of causes, but infectious etiologies are particularly noteworthy. The sixth cranial nerve is particularly susceptible to injury due to its long intracranial course, and infections are a recognized cause of cranial neuropathy. Common infectious agents in pediatric age include *Mycoplasma pneumoniae*, *Chlamydia pneumoniae*, and HSV, which have been linked to several central nervous system (CNS) manifestations [10–12].

Acquired ANP in children may be the presenting feature of *Mycoplasma pneumoniae* or *Chlamydia pneumoniae* infection [13–16]. While meningoencephalitis and acute disseminated encephalomyelitis are more common, there have been instances of isolated ANP associated with *Mycoplasma pneumoniae* in children [13]. One such case involved a 26-month-old boy who developed bilateral ANP, which was attributed to *Mycoplasma pneumoniae* infection [14]. There is limited literature regarding the role or association of *Chlamydia pneumoniae* with ANP. Eijnden et al. (2001) reported a case of isolated ANP in a child associated with asymptomatic *Chlamydia pneumoniae* infection and elevated anti-GQ1b antibodies, suggesting that even asymptomatic infections can trigger significant neurological response [16]. The exact pathogenetic mechanism behind CNS involvement associated with these infections is still not fully understood, and more research is required. Given their relatively high occurrence, clinicians must be vigilant about their wide range of possible presentations, including ANP, even when typical respiratory symptoms are absent [15].

HSV is a significant cause of infections worldwide, with HSV-1 and HSV-2 leading to various clinical manifestations in children and adolescents. One rare but notable complication might be the involvement of the abducens nerve. A recent documented case involved a young immunocompetent female presenting with meningoencephalitis and ANP due to a primary HSV-2 infection. The authors proposed primary cranial neuritis or perineuritis from meningoencephalitis as the underlying mechanism, given the unilateral nature of the palsy, a normal brain MRI and no significant elevation in intracranial pressure [17]. HSV-2 should, therefore, be suspected even in the absence of genital symptoms. HSV-1 has also been hypothesized to play a role in idiopathic cranial nerve palsies, including ANP. PCR analysis of CSF in a 68-year-old woman with fourth and sixth nerve palsy confirmed the presence of HSV-1 DNA; intravenous acyclovir treatment led to resolution within seven weeks [18]. Furthermore, research investigated the presence of HSV-1 in various cranial nerve nuclei, including the abducens nerve nucleus: the findings indicated that HSV-1 DNA was randomly distributed across all examined cranial nerve nuclei and adjacent control tissues, including the abducens nerve nucleus, suggesting that HSV-1 was not specifically localized to the abducens nerve nucleus, thus challenging the hypothesis that HSV-1 reactivation in this specific nucleus could be responsible for ANP [19]. PCR is now considered the gold standard for diagnosing HSV infections in the CSF and blood of children, even when serum IgM antibodies are absent. This is partly due to the limited time frame during which IgM antibodies are detectable and their lower sensitivity and specificity compared with PCR [20]. In our patient, the pathogenic role of HSV appears limited, given the negative PCR results and the isolated IgM positivity.

*Borrelia burgdorferi*, the causative agent of Lyme disease, is known for its wide range of neurological manifestations, including facial nerve palsy and meningoradiculitis [21]. However, isolated ANP is a less common presentation. Studies have shown that *Borrelia burgdorferi* can have a crucial role in a significant proportion of Bell's palsy cases, suggesting that Lyme disease should be considered in the differential diagnosis of facial nerve palsy in endemic areas [22]. Cases of Lyme disease presenting with isolated ANP, or in combination with other cranial nerves, have been reported in the literature, both in pediatric and adult populations [23–28]. Patients typically present with symptoms such as painful diplopia and may have a history of tick exposure or erythema migrans [29]. Moreover, pathological cranial nerve MRI enhancement has been reported in up to 57% of neuroborreliosis cases. Although abducens nerve enhancement did not appear to correlate with eye movement palsy, Lyme neuroborreliosis should still be considered in the differential diagnosis in cases of pathological cranial nerve enhancement, as done in our case, regardless of the presence or absence of clinical signs of neuropathy [30].

The therapeutic approach in our case combined corticosteroids with antibiotics and antiviral therapy, targeting the suspected pathogens. Although serological findings alone did not confirm active infection, the patient's gradual recovery over a few months suggested that the treatment had addressed an underlying infectious or postinfectious inflammatory process. This empirical “shotgun” approach reflects common clinical practice in uncertain scenarios, aiming to cover multiple possible infectious etiologies while modulating inflammation.

Spontaneous recovery from ANP is influenced by various factors, including the underlying cause, whether it is unilateral or bilateral, and the extent of abduction limitation [31]. Complete recovery in third, fourth, and sixth cranial nerve palsies has been associated with an onset of less than 7 days and isolated palsy [32]. This raises the question of whether the therapeutic interventions directly influenced the resolution or whether the outcome reflects the natural history of the condition. Further studies are required to establish definitive causal relationships and clarify the role of targeted treatments in similar cases.

A multidisciplinary approach involving neurology, infectious disease, and ophthalmology specialists is necessary. Repeated clinical and radiological evaluations ensured that potentially serious underlying conditions, such as neoplasms or intracranial hypertension, were excluded. MRI, in particular, played a crucial role in ruling out structural abnormalities. Given the wide range of potential etiologies, this case highlights the diagnostic complexity involved when initial investigations fail to identify a clear underlying pathology. The mild IgM positivity for HSV-1, *Mycoplasma pneumoniae*, *Chlamydia pneumoniae*, and *Borrelia burgdorferi* provided a potential, though not definitive, explanation for the patient's condition, emphasizing the challenges of interpreting serological results in such scenarios. This case also highlights the broader limitations of isolated IgM serology. Low-positive IgM results, especially when not accompanied by IgG seroconversion or positive PCR, are prone to false positivity due to cross-reactivity and the prolonged persistence of IgM antibodies. These pitfalls reduce the positive predictive value of such findings and can lead to unnecessary concern or treatment. Therefore, isolated IgM seropositivity should be interpreted with great caution and always in the context of clinical features and more reliable diagnostic tools.

In conclusion, this case underscores that in pediatric ANP: (1) clinicians should promptly prioritize neuroimaging and CSF analysis to exclude urgent or life-threatening causes; (2) isolated, low-positive IgM results should be interpreted with extreme caution, as they often lack diagnostic significance in the absence of IgG seroconversion or PCR confirmation; and (3) a benign postinfectious inflammatory neuritis remains a likely explanation in such cases, which typically have an excellent prognosis for spontaneous recovery. This practical approach may help avoid misdiagnosis and unnecessary interventions.

## Data Availability

Data sharing is not applicable to this article as no new data were created or analyzed in this study.
